# COVID-19: update of the Italian situation

**DOI:** 10.1007/s13365-020-00900-w

**Published:** 2020-09-08

**Authors:** Carla Prezioso, Valeria Pietropaolo

**Affiliations:** 1 Microbiology of Chronic Neuro-degenerative Pathologies, IRCSS San Raffaele Pisana, Rome, Italy; 2 Department of Public Health and Infectious Diseases, “Sapienza” University, P.le Aldo Moro, 5, 00185 Rome, Italy

**Keywords:** COVID-19, SARS-CoV-2, COVID-19 new cases and deaths, Geographical distribution, Lockdown

## Abstract

On the March 11, 2020, the World Health Organization (WHO) declared the novel coronavirus disease 2019 (COVID-19) outbreak as a pandemic. The first cases in Italy were reported on January 30, 2020, and quickly the number of cases escalated. On March 20, 2020, according to the Italian National Institute of Health (ISS) and National Institute of Statistics (ISTAT), the peak of COVID-19 cases reported in Italy reached the highest number, surpassing those in China. The Italian government endorsed progressively restrictive measures initially at the local level, and finally, at the national level with a lockdown of the entire Italian territory up to 3 May 2020. The complete Italian territory closing slowed down the contagion. This review retraces the main numbers of the pandemic in Italy. Although in decline, the new reported cases remain high in the northern regions. Since drugs or vaccines are still not available, the described framework highlights the importance of the containment measures to be able to quickly identify all the potential transmission hotspots and keep control subsequent epidemic waves of COVID-19.

The coronavirus 2019 (COVID-19) is an infectious disease caused by severe acute respiratory syndrome coronavirus 2 (SARS-CoV-2), which was declared as a global public health emergency and affected all countries, all over the world, from the beginning of the year 2020 (Prezioso et al. 2020; Zhu et al. 2020). On the March 11, 2020, the World Health Organization (WHO) declared this ongoing outbreak as a pandemic (WHO Coronavirus disease 2019, situation report - 51).

Italy was the first European country to be severely affected: by March 30, there were more than 101,739 people who tested positive for SARS-CoV-2 (Italian Ministry of Health Daily Bulletin Covid-19 Outbreak in Italy 2020). Cases were more common among women (53.1%), mainly among the elderly population but lethality is higher in male subjects with comorbidities (Italian National Institute of Health (ISS) and National Institute of Statistics (ISTAT) (May, 4th) 2020a), as shown previously in China (Wu and McGoogan 2020). The median age at COVID-19 diagnosis was 62 years old. In Italy, geographical spread of the COVID-19 has been heterogeneous with the highest spread in the Northern regions and the lowest in the Southern regions and in the main Islands. The region of Lombardy has had the highest number of SARS-CoV-2’s cases and appears to be the epicenter of the Italian outbreak (Italian National Institute of Health (ISS) and National Institute of Statistics (ISTAT) (May 4) 2020a). In fact, up to 30 March, among 101,739 people tested positive for SARS-CoV-2, 42,161 positive people (41%) were detected only in Lombardy (Fig. 1a). Thirteen thousand five hundred thirty-one (13,531) cases were detected in Emilia Romagna followed by 8724 cases in Veneto, 8712 cases in Piedmont, 4412 cases in Tuscany, 3684 cases in Marche region, 3217 cases in Liguria, 3007 cases in Trentino Alto Adige (Trento and Bolzano), 2914 cases in Lazio, 1952 cases in Campania, 1712 cases in Apulia, 1555 in Sicily, 1501 cases in Friuli Venezia Giulia, 1345 cases in Abruzzo, 1051 cases in Umbria, 682 cases in Sardinia, 647 cases in Calabria, 584 cases in Val d’Aosta, 214 cases in Basilicata and, finally, by 134 cases in Molise (Italian Ministry of Health, Daily Bulletin Covid-19 Outbreak in Italy 2020) (Fig. 1a). On 30 March, the total number of deaths, at a national level, was 11,591 of which 6818 only in Lombardy (59%), 1538 in Emilia Romagna (13%), 749 in Piedmont (6.2%), 417 in Marche region (3.6%), 413 in Veneto (3.6%), 397 in Liguria (3.4%), 231 in Tuscany (2%), 221 in Trentino Alto Adige (Trento and Bolzano) (2%), 150 in Lazio (1.3%), 125 in Campania (1%), 107 in Friuli Venezia Giulia (0.9%), 102 in Abruzzo (0.8%), 91 in Apulia (0.8%), 76 in Sicily (0.6%) and less than 50 in the other five regions (≤ 0.4%) (Italian Ministry of Health Daily, Bulletin Covid-19 Outbreak in Italy 2020) (Fig. 1c). The lethality rate increased with age and was higher in males versus females: 0% from 0 to 29 and 0.3% between 30 and 49 years of age; 1.9% in the age group 50–59 years (0.8% in women and 2.4% in men); 6.4% from 60 to 69 years (3.5% in women and 6.9% in men); 18.5% from 70 to 79 years (11.7% in women and 19.8% in men); 26.2% from 80 to 89 years (18.9% in women and 29.2% in men) and 24.8% after 90 years of age (20.4% in women and 30.8% in men) (Distante et al. 2020, Italian Ministry of Health Daily, Bulletin Covid-19 Outbreak in Italy 2020).

**Fig. 1:**
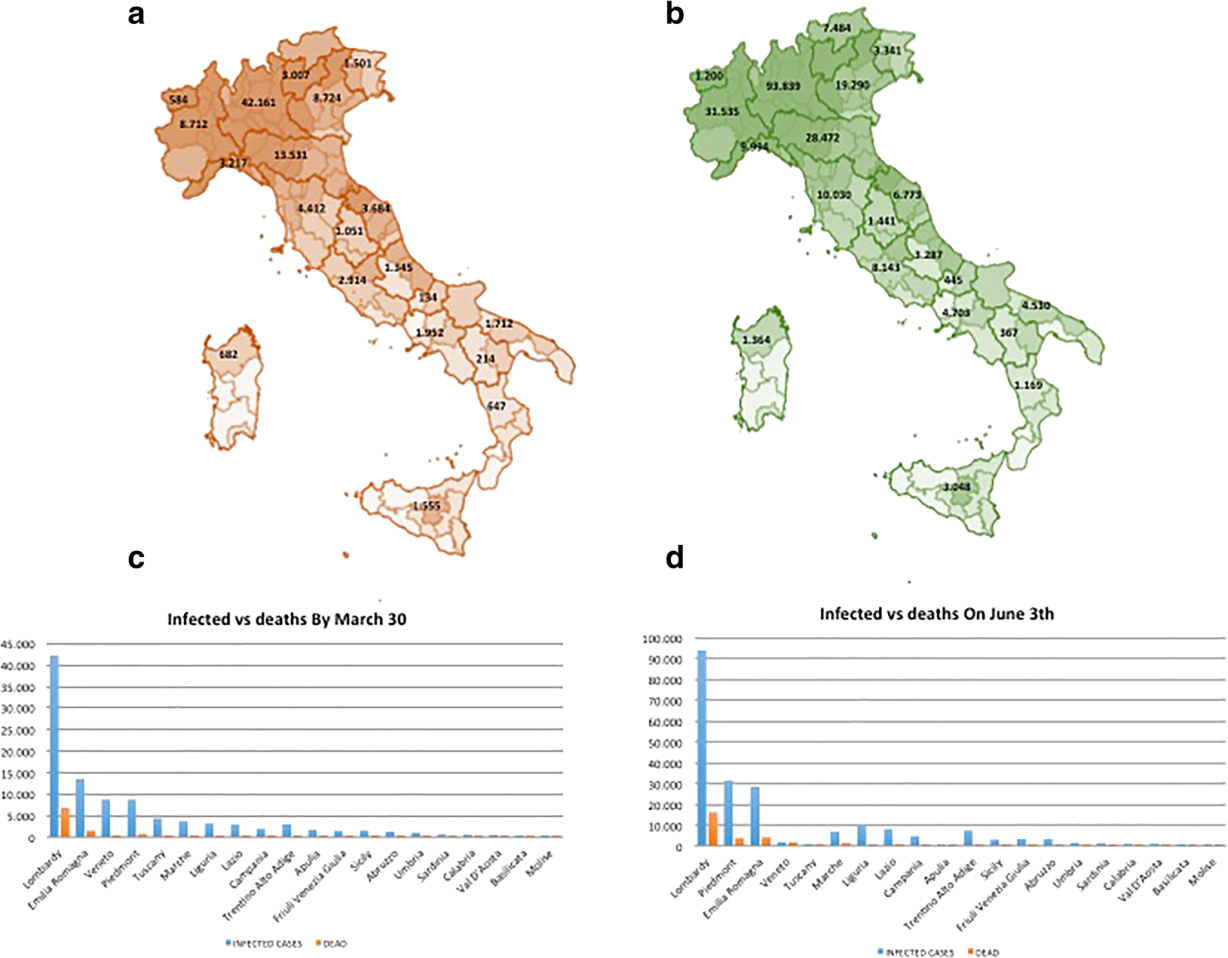
The geographical distribution of COVID-19 cases and associated deaths in Italy during the pre- and post-lockdown period of the epidemic. **a** During the pre-lockdown period, the highest geographical SARS-CoV-2 spread was reported in the northern regions of Italy and the lowest in the southern regions and in the main Islands. The region of Lombardy has the highest number of cases of SARS-CoV-2 and appears to be the epicenter of the Italian outbreak. **b** In the post-lockdown period, the heterogeneity in the geographical spread of the epidemic was confirmed and the SARS-CoV-2 circulation persisted high in the Northern regions and held in the Southern Regions and in the Islands. **c** On 30 March, the total number of deaths, at a national level, was 11,591 of which 6818 only in Lombardy, 1538 in Emilia Romagna, 749 in Piedmont, 417 in Marche region, 413 in Veneto, 397 in Liguria, 231 in Tuscany, 221 in Trentino Alto Adige (Trento and Bolzano), 150 in Lazio, 125 in Campania, 107 in Friuli Venezia Giulia, 102 in Abruzzo, 91 in Apulia, 76 in Sicily and less than 50 in the other five regions. **d** On June 3, the total number of deaths was 33,601 of which 16,172 in Lombardy. Three thousand eight hundred ninety-eight (3898) deaths were reported in Piedmont, 4147 in Emilia Romagna, 1921 in Veneto, 1473 in Marche region, 1055 in Tuscany, 987 in Lazio, 754 in Trentino Alto Adige (Trento and Bolzano), 747 in Liguria, 511 in Apulia, 415 in Campania, 414 in Abruzzo, 336 in Friuli Venezia Giulia, 275 in Sicily, 143 cases in Calabria, 131 cases in Sardinia, 97 cases in Val d’Aosta, 76 cases in Umbria, 27 cases in Molise and, finally, by 22 cases in Basilicata

According to the Italian National Institute of Health (ISS) and National Institute of Statistics (ISTAT), the peak of COVID-19 cases reported in Italy reached the highest number on March 20; therefore, it started to decrease. In April, 94,257 cases were reported (Italian National Institute of Health (ISS) and National Institute of Statistics (ISTAT) (June 4) 2020b), although many deaths referred to those were diagnosed in March. The decline continued sharply in May when 22.893 cases were described (Italian National Institute of Health (ISS) and National Institute of Statistics (ISTAT) (July 9) 2020c).

The reduction in the number of COVID-19 diagnosis is attributable to several preventive measures that were undertaken to favour “social distancing”, initially at the local level, and finally, at the national level with a lockdown of the entire Italian territory up to 3 May 2020 (Italian Ministry of Health COVID-19, Gazzetta Ufficiale Decreto #Iorestoacasa (March 10) 2020). The complete closing slowed down the contagion and, by May 31, 232,639 cases of COVID-19 and 32,981 associated deaths were detected (Italian National Institute of Health (ISS) and National Institute of Statistics (ISTAT) (July 9) 2020c). The heterogeneity in the geographical spread of the epidemic was confirmed and the SARS-CoV-2 circulation persisted high in the Northern regions and held in the Southern Regions and in the Islands. Considering COVID-19 cases and deaths, 75% of the reported cases and 82% of the deaths were in the provinces defined as “high” spread, 17% of the cases and 13% of the deaths in the “medium” spread and respectively 8% and 5% in the provinces with “low” spread (Italian National Institute of Health (ISS) and National Institute of Statistics (ISTAT) (July 9) 2020c). The mortality of the high-spread provinces decreased from 44,998 in March 2020 (113.1% more than in 2015–2019) to 32,931 in April (73.9% more than in 2015–2019). The most important drop was observed in Lombardia: deaths for the overall causes decreased from 24,893 in March to 16,190 in April 2020. The provinces most affected by the epidemic were the ones in which the most important reductions were observed. In Bergamo and Lodi, the mortality decreased from 571% in March to 123% in April and from 377 to 79.9%, respectively. The excess mortality in March and April 2020 was more significant for men aged 70–79 and 80–89 years, for whom the cumulative deaths, from January 1 to April 30, 2020, increased by more than 52 percentage points, compared with the same period of the 2015–2019 average. For people aged 90 and over, excess mortality had an increase of 48%. The increase in female mortality was more contained for all age classes (Italian National Institute of Health (ISS) and National Institute of Statistics (ISTAT) (June 4) 2020b).

On June 3, the rigorous lockdown measures to contain the epidemic were loose and the inter-region movements were allowed again. The movement between regions caused a crucial challenge. Among 240,455 people tested positive for SARS-CoV-2, 93,839 positive people (39%) were detected only in Lombardy (Fig. 1b). Thirty-one thousand five hundred thirty-five (31,535) cases were detected in Piedmont, followed by 28,472 cases in Emilia Romagna, 19,290 cases in Veneto, 10,030 cases in Tuscany, 9994 cases in Liguria, 8143 cases in Lazio, 7484 cases in Trentino Alto Adige (Trento and Bolzano), 6773 cases in Marche region, 4703 cases in Campania, 4530 cases in Apulia, 3341 cases in Friuli Venezia Giulia, 3287 cases in Abruzzo, 3048 cases in Sicily, 1441 cases in Umbria, 1364 cases in Sardinia, 1200 cases in Val d’Aosta, 1169 cases in Calabria, 445 cases in Molise and, finally, by 367 cases in Basilicata (Italian National Institute of Health (ISS) COVID-19 monitoring report (June 19–30) 2020) (Fig. 1b). On June 3, the total number of deaths was 33,601 of which 16,172 in Lombardy (48%). Three thousand eight hundred ninety-eight (3898) deaths were reported in Piedmont, 4147 in Emilia Romagna, 1921 in Veneto, 1473 in Marche region, 1055 in Tuscany, 987 in Lazio, 754 in Trentino Alto Adige (Trento and Bolzano), 747 in Liguria, 511 in Apulia, 415 in Campania, 414 in Abruzzo, 336 in Friuli Venezia Giulia, 275 in Sicily, 143 cases in Calabria, 131 cases in Sardinia, 97 cases in Val d’Aosta, 76 cases in Umbria, 27 cases in Molise and, finally, by 22 cases in Basilicata (Italian National Institute of Health (ISS) COVID-19 monitoring report (June 19–30) 2020) (Fig. 1d).

During the period between 15 and 28 June, the general picture of the transmission and impact of SARS-CoV-2 infection in Italy continued to be low critical with a cumulative incidence of 4.7 per 100,000 inhabitants, with national Rt index < 1 (Italian Ministry of Health COVID-19 weekly monitoring report (June 22–28) 2020). A slight increase in the number of newly diagnosed cases was described in the week of July 6–13 with respect to the previous week of monitoring with Rt reproduction number of 1.01 (Italian Ministry of Health COVID-19 weekly monitoring report (June 29–July 12) 2020). It is likely that for many of the reported cases, the infection was contracted 2–3 weeks earlier, so prevalently in the last 10 days of June. To date, some small chains of transmission of unknown origin continue to be disclosed on the national territory and in addition, some outbreaks are attributable to an increase in imported cases from other regions or foreign states. This highlights that the COVID-19 epidemic in Italy is not over. The Rt estimates continue to fluctuate in some regions and in the autonomous provinces as Trento and Bolzano, in relation to outbreaks of transmission hotspots that are subsequently contained (Italian Ministry of Health COVID-19 weekly monitoring report (June 29–July 12) 2020). As a result, in the last 14 days (week of July 13–19), a national Rt number of 0.95 has been observed indicating that transmission in our country is substantially stationary. Although in decline, the number of new cases reported remains high in six regions with estimates of Rt rates of over 1 (Italian Ministry of Health COVID-19 weekly monitoring report (July 13–19) 2020). This calls for caution as it shows that, in some parts of the country, SARS-CoV-2 is still circulating and when favourable conditions occur, outbreaks can arise. However, currently, at the national level, there is a stable transmission of the virus. This is thanks to testing-tracing-tracking activities, which make it possible to break potential transmission chains at an early stage. The reduction in the time between the start of symptoms and diagnosis/isolation makes faster identification and clinical care for the people who contract the infection possible. It is not surprising to observe a low number of cases that require hospitalization as, given the characteristics of the COVID-19 illness, only a small proportion of the total number of people who contract the SARS-CoV-2 virus go on to have a serious clinical situation. In part this is due to the characteristics of the outbreak, which see a less involvement of elderly people, since in recent weeks, the average age of the cases diagnosed is around 40. This outcome, which is expected because of the strategy adopted during the lockdown phase, makes it possible to manage the presence of the virus on the national territory, in a situation of reopened activities, without overloading health care services.

Since drugs or vaccines are still not available, it is essential to keep attention levels high and continue to strengthen testing-tracing-tracking activities in order to be able to quickly identify all the potential transmission hotspots and keep control of the epidemic. Otherwise, we could see a reversal of the trend in the coming weeks, with subsequent epidemic waves of COVID-19 a significant increase in the number of cases, at the national level. For this reason, it is necessary to keep awareness high about the fluidity of the epidemiological situation and the importance of continuing to rigorously respect of the measures necessary to reduce the risk of transmission, such as individual hygiene and physical distancing. It is important for people to self-isolate “in quarantine if coming from high-incidence countries”.

## Data Availability

No datasets were generated during the study.

## Abbreviations

COVID-19: Coronavirus disease 2019
ISS: Italian National Institute of Health
ISTAT: National Institute of Statistics
SARS-CoV-2: Severe acute respiratory syndrome coronavirus 2
WHO: World Health Organization
